# International observatory on mental health systems: structure and operation

**DOI:** 10.1186/1752-4458-3-8

**Published:** 2009-04-03

**Authors:** Harry Minas

**Affiliations:** 1 Centre for International Mental Health, Melbourne School of Population Health, The University of Melbourne, Parkville, Victoria 3010, Australia

## Abstract

**Introduction:**

Sustained cooperative action is required to improve the mental health of populations, particularly in low and middle-income countries where meagre mental health investment and insufficient human and other resources result in poorly performing mental health systems.

**The Observatory:**

The International Observatory on Mental Health Systems is a mental health systems research, education and development network that will contribute to the development of high quality mental health systems in low and middle-income countries. The work of the Observatory will be done by mental health systems research, education and development groups that are located in and managed by collaborating organisations. These groups will be supported by the IOMHS Secretariat, the International IOMHS Steering Group and a Technical Reference Group.

**Summary:**

The International Observatory on Mental Health Systems is: 1) the mental health systems research, education and development groups; 2) the IOMHS Steering Group; 3) the IOMHS Technical Reference Group; and 4) the IOMHS Secretariat. The work of the Observatory will depend on free and open collaboration, sharing of knowledge and skills, and governance arrangements that are inclusive and that put the needs and interests of people with mental illness and their families at the centre of decision-making. We welcome contact from individuals and institutions that wish to contribute to achieving the goals of the Observatory.

*Now is the time to make it happen where it matters, by turning scientific knowledge into effective action for people's health*. (J.W. Lee, in his acceptance speech on his appointment as the Director-General of the World Health Organization) [1].

## Introduction

Sustained cooperative action is required to improve the mental health of populations everywhere [2]. This is particularly so in low and middle-income countries where mental health system performance is poor as a result of meagre mental health investment [3,4] and strikingly insufficient and inequitably distributed human and other resources [5,6]. While many aspects of health and illness are universal, they are imbedded, and must be understood, in local socio-cultural, economic and political contexts. Such understandings can only be gained through high quality locally relevant research.

The particularities of local problems require the development of locally relevant and effective solutions. This requires strong local capacity in all relevant domains. One such domain, currently poorly developed, is mental health systems research. In most low and middle-income countries there is limited capacity to answer critical questions about the mental health status and needs of populations, how mental health is most effectively maintained and illness prevented, which treatments are effective, whether population health policy objectives are being met, and how health systems are most effectively organised. If the mental health of populations is to be improved such questions must be answered [7]. They can only be meaningfully answered through local research that is high quality and is carried out by local researchers.

## The Observatory

**Figure 1:**
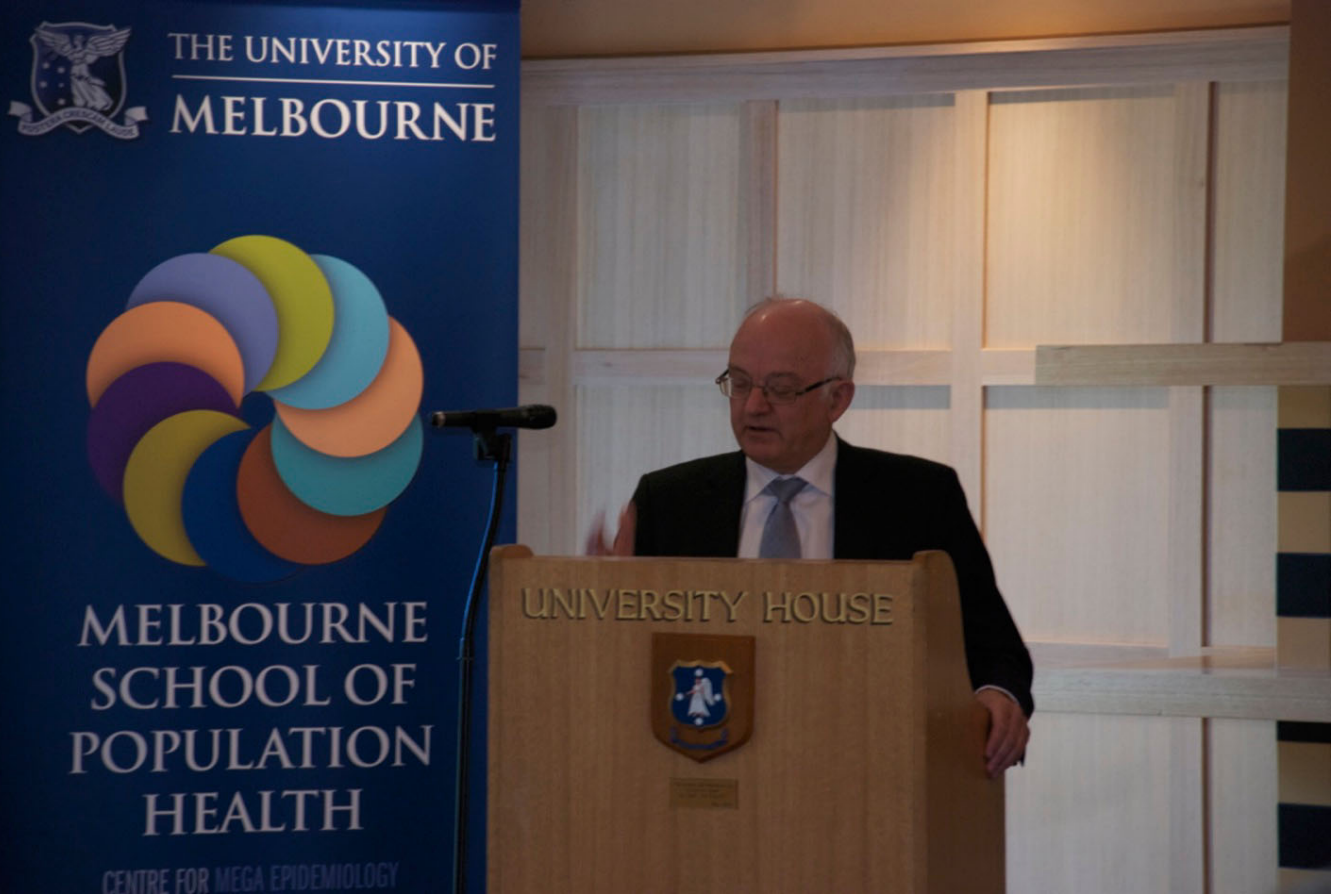
**The Hon Bob McMullan, Australian minister for international development assistance, launching the Observatory on 6 February 2009**.

The International Observatory on Mental Health Systems [8] was formally launched on 6 February 2009 at the University of Melbourne by The Hon Bob McMullan, Australian Minister for International Development Assistance (Figure 1). In his speech Mr McMullan made the following observations.

"The observatory will be an important source of information for governments who want to develop good mental health policies and best practices. Mental illness is common in all countries. Treatment and care is complex. Community attitudes and stigma surrounding mental illness take time to change. We are a developed country and we are still on a learning curve trying to get it right. Several countries in Asia and the Pacific are seeking to build up their capacity in mental health. The research you do will be critical in helping them improve their mental health services. I am a firm believer in the need for good evidence-based policy and programs. One of the key findings during consultations for the Development for All strategy [9], was the dearth of both quantitative and qualitative information about disability in our region. Governments and policy makers everywhere need good, sound information on which to make decisions. Your work will make a valuable contribution."

The goals of the Observatory are to:

• foster the establishment, and support, a vibrant mental health systems research, education and development network, that will produce evidence for mental health policy and practice in low and middle-income countries, and build capacity for mental health system development;

• produce new knowledge to inform the development of mental health systems that are effective, accessible, equitable, culturally appropriate, affordable and disability inclusive, and that protect the human rights of people with mental illness, and

• monitor and evaluate progress in the development of mental health systems in low and middle-income countries.

**Figure 2:**
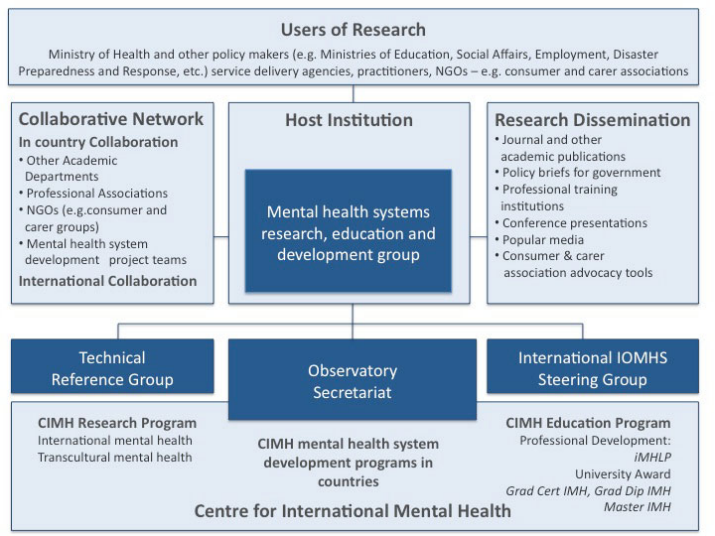
**Structure of the International Observatory on Mental Health Systems**.

The structure of the Observatory is shown in Figure 2. The Observatory is those sections of Figure 2 coloured in blue. The rest of the figure illustrates a pattern of collaborations. At the heart of the Observatory, as it develops, will be the mental health systems research, education and development groups, established and managed by collaborating institutions. It is envisaged that these groups, initially brought together from existing staff and augmented with new positions when these can be funded, will carry out a coherent program composed of mental health systems research, education and development projects. In those academic institutions where resources and local arrangements permit, the formal establishment of centres for mental health systems research, education and development will be encouraged and supported.

The IOMHS Secretariat, located in the Centre for International Mental Health, will play a coordinating role and will support the International Steering Group, the Technical Reference Group, and collaborating groups.

An International IOMHS Steering Group has been established, with members from Australia, Cambodia, China, Egypt, Ghana, India, Indonesia, Japan, Malaysia, New Zealand, Pakistan, Philippines, South Africa, Sri Lanka, Switzerland, Thailand, UK, USA and Vietnam. The Steering Group will be expanded to ensure that all countries in which the Observatory has active programs are represented. The role of the Steering Group is to ensure that governance of the Observatory is representative, transparent and accountable.

The IOMHS Technical Reference Group will advise on technical aspects of research design, data collection, analysis and interpretation, research ethics, research governance and publication of research results. The membership of the Group will be researchers with high level technical skills who wish to contribute to building mental health systems research capacity in low and middle-income countries, particularly by supporting more junior research colleagues to develop their skills.

## IOMHS collaborating organisations

"The work program of IOMHS will depend on the establishment of partnerships with governments, universities, international and local NGOs and other partner organisations that will collect, analyse and apply high quality information for mental health system reform and development. An outcome of such a collaborative work program will be the establishment of sustained and productive collaborations between mental health policy makers, academics, practitioners, NGOs, and civil society organisations, and improved capacity to analyse trends in health care reform. An explicit goal is to build the strengths of partner organisations and networks to provide evidence-based advice to national and sub-national policy makers, service planners and implementers." [8]

The major part of the work of the Observatory will be done by collaborating partners in participating countries. Collaborating organisations may be Ministries of Health, Universities, local and international NGOs, and civil society organisations – particularly consumer and carer groups. It is anticipated that, in any one country, a number of organisations of different kinds will enter into collaborative agreements to form and enlarge IOMHS. While all such collaborations will be welcomed by IOMHS a core strategy in building these collaborations will be the establishment of mental health systems research, education and development groups and, as resources and circumstances permit, some institutions may establish formal centres for mental health systems research, education and development. It is expected that these groups will:

• be located in an appropriate academic department or other organisation;

• have a close working relationship with the Ministry of Health and other relevant government ministries (e.g. Education, Social Affairs, Employment, etc.) and actively support the use of research findings for evidence-based mental health system development;

• be committed to establishing and strengthening collaborative working relationships with mental health stakeholder groups in the country (e.g. national professional associations, other universities, public and private mental health service agencies, and NGOs – particularly associations of consumers and carers), and with groups with similar goals internationally;

• be committed to education and training in mental health systems research and in policy and service development;

• be committed to the ethical conduct of mental health systems research and, where they do not formally exist, the establishment of ethics review procedures for such research;

• be disability inclusive;

• promote and support capacity development in consumer and carer organisations and facilitate and support consumer-led and carer-led research;

• be committed to open access publishing to ensure that the results of the work of the Observatory are freely available to all;

• share what they learn through participation in the annual International Mental Health System Development Conference and other relevant national and international meetings.

The work agenda and priorities of such groups will, of course, be a matter for the groups themselves to determine, on the basis of local and national needs and priorities. With the support of IOMHS such groups will build the opportunities to participate in or to lead multi-country collaborative research projects on matters that are of regional and global as well as local significance.

## Research capacity strengthening

What is to be strengthened? Research capacity is multidimensional. It includes the production, dissemination and use of knowledge. It includes the ability – the existence of the necessary skills and resources -and the motivation to produce, widely disseminate and appropriately use newly acquired knowledge. It includes also systems for allocating priority, or value, to different research objectives, to ensure that research contributes to national health goals.

Dyson and Desforges [10] have suggested strategies for strengthening research capacity. The Observatory aims to implement each of these strategies in the following ways (See Figure 2);

### Resource concentration

Available resources can be concentrated in particular institutions, teams, and networks that can focus on national health priorities in order to enable an increased volume of high-quality and well-targeted research to be undertaken.

A central element of the Observatory strategy is resource concentration through the establishment of mental health systems research, education and development groups and, where possible, formal centres committed to such work. This will bring together and support researchers with an interest and expertise in mental health systems research and will continuously build such expertise.

### Networking

Improving interaction between different parts of the research system so that available resources are used effectively and efficiently, with the additional benefit that interaction may produce a 'multiplier' effect. The networks should be across different institutions and teams, across different parts of the research system (e.g. research producer and user groups) and across different disciplines.

A key role of the collaborating organisations is to build such networks in the country in which they are located and to establish collaborations with centres in other countries in order to facilitate knowledge exchange.

### Communication infrastructure

Well-functioning communication infrastructure is essential.

The IOMHS Secretariat will develop effective means for communication within the IOMHS network, and between IOMHS and other organisations.

### User-researcher interaction

When it is intended that the research should influence policies and practice, it is essential that the users of the research results are engaged as early as possible in the research process. This will improve commitment by users to the research, will ensure that the research is relevant to the goals of the users, and will make it more likely that the research results are understood and used in an appropriate manner.

IOMHS collaborating groups should have established links with key research users, particularly Ministries of Health and other relevant government agencies, as well as other research user organisations. A particularly important responsibility of the collaborating oranisations in the IOMHS network is that research outcomes are turned into tools for advocacy, for use by consumer and carer organisations and by other civil society organisations, and into policy briefs, for use by policy makers and implementers.

### Strategic leadership

This involves taking a systemic view of research capacity and creating mechanisms for formulating and implementing strategies for its development.

Within countries the IOMHS collaborating groups will take primary responsibility for strategic leadership. They will particularly have responsibility for shaping the national mental health systems research agenda. They will be supported in this role by the IOMHS Steering Group, in which all IOMHS collaborating groups will be represented, the Technical Reference Group, and the IOMHS Secretariat.

### Training and skills development

Those who are engaged in research must have the necessary skills if they are to carry out research confidently and well, and if they are to maintain a commitment to research. There are many forms that such training can take, such as basic skills development, research higher degrees and training in leadership for research. Often neglected but very important is training in research governance and research ethics.

**Figure 3:**
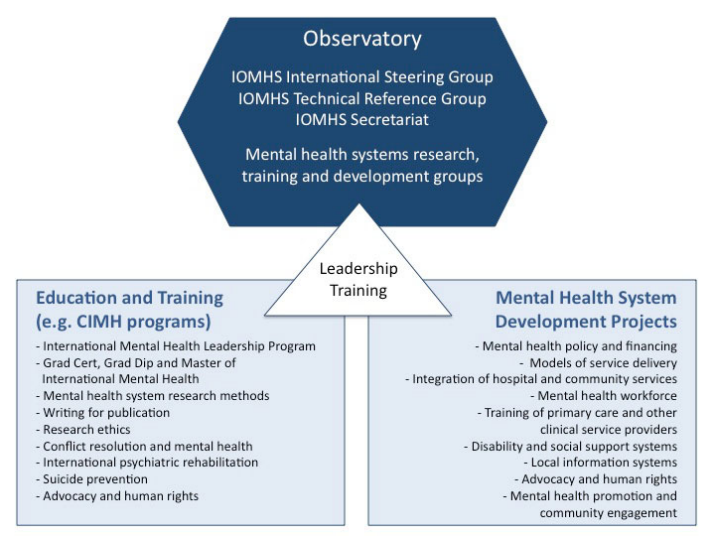
**Links between the work of the Observatory, education and training and mental health system development**.

**Figure 4:**
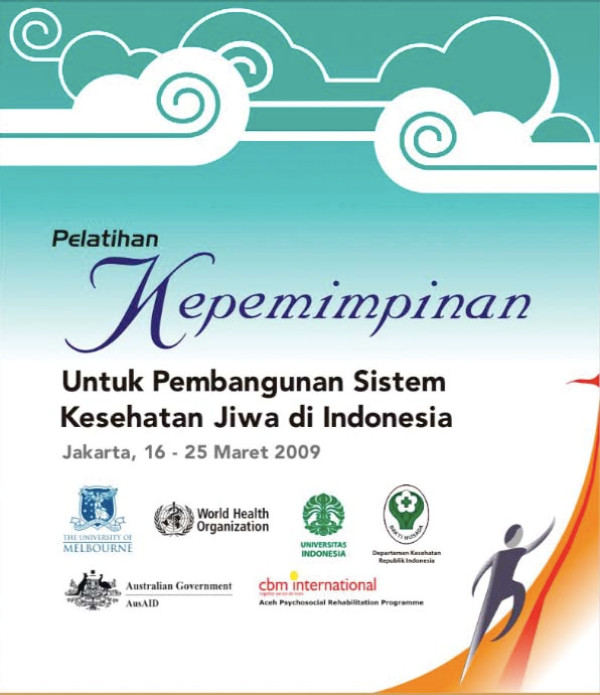
**Leadership for Mental Health System Development training program offered by the National Taskforce for Mental Health System Development in Indonesia, Jakarta, 16–25 March 2009**.

Observatory partners will be explicitly linked into training opportunities, such as the professional development (International Mental Health Leadership Program) and award programs (Graduate Certificate, Graduate Diploma and Master of International Mental Health) offered by the Centre for International Mental Health [11,12] (Figure 3), and relevant training programs offered by other organisations, such as the International Master in Mental Health Policy and Services [13] and the International Diploma in Mental Health Law and Human Rights [14]. The Observatory will specifically work with IOMHS collaborating groups to support the development and delivery of priority training programs in the countries and in the languages in which the IOMHS collaborating groups work, such as the Leadership for Mental Health System Development in Indonesia training program [15] (Figure 4). An important focus of the training opportunities will be leadership training [11], since the strengthening of leadership for mental health system development is a fundamental objective of the Observatory. Examples of other training programs to be offered include: mental health systems research methods, writing for peer-reviewed journal publication and research ethics. Graduate Certificate, Graduate Diploma and Master of International Mental Health courses are also available for those who wish to pursue formal academic qualifications.

### Research career development

Training is one component may form part of a wider attempt to open up career pathways which encourage talented individuals to become involved in research and then to develop their skills to the highest possible level.

The establishment of mental health systems research, education and development groups in key academic departments will open up career pathways that currently do not exist for mental health systems researchers. Increased expertise and the existence of groups with the benefits of scale will improve ability to compete for scarce research funds from national and international sources, further enhancing technical capacity and career opportunities. Professional development courses and formal university qualifications in international mental health will make a substantial contribution to building careers in mental health systems development and will support career advancement.

### Ensuring political independence

Establish mechanisms for the management of research that enables activity to be directed by a concern for knowledge-building rather than by short-term political expediency. Whilst research should inform policy, political micro-management of research is likely to be damaging to capacity rather than otherwise.

The expectation that most IOMHS collaborating groups will be established in influential academic institutions, which everywhere jealously guard their independence, will contribute to ensuring political independence. However, political independence does not mean political isolation. Unless such research is respected and used by those who are directly engaged in the political process it is unlikely to have any influence on, or to contribute to, mental health system development. An important element in ensuring political independence is the strengthening of a culture of research (see below).

### Cultural change

A key component of cultural change is the creation of a culture in which the production, dissemination and use of high-quality research is valued as an integral part of the health system.

National research capacity is a reflection of national culture. Culture consists of values, beliefs, practices, language and systems of power. Cultures are most clearly expressed in the patterns of relationships between individuals and in the institutions that a culture creates. Some of the beliefs concerning knowledge that are implicit in most research are that knowledge is created or discovered rather than 'received' or 'revealed'. It is provisional, always open to change in the face of new evidence. The beliefs held or the knowledge promoted by authoritative individuals and institutions are not exempt from scrutiny and challenge that is made necessary by evidence. Knowledge should be publicly owned, widely disseminated and shared, and should be used for public benefit. Such beliefs may be considered dangerous, and may be suppressed, in settings where religious, social, economic, political orthodoxies prevail. Any such suppression is inimical to high quality research. Table 1 sets a possible set of research values.

**Table 1:** Research values

**Excellence**: Ensure that research is of the highest quality
**Curiosity**: Encourage curiosity and support exploration
**Free inquiry**: Remove impediments to free inquiry
**Attitudes to authority**: Examine and question authority
**Justice and equity**: Ensure that research contributes to the public good
**Research ethics**: Protect and enhance the rights and interests of research participants by establishing processes that ensure ethical research practice

### Resource maximisation

Increasing the resources available within the research system (which ultimately means increasing the funding dedicated to research) is the most obvious means of strengthening capacity. However, careful attention should also be paid to the coordinated and efficient use of existing resources.

"Funding for health systems research in developing countries and by developing country researchers is meagre. Evidence suggests that such funding is at most 0.02% of health expenditure, far too low to have an impact on health system development. Funding should be mobilized from within national health systems as well as from science and technology budgets and international sources. Priority problems for research need to be identified in relation to health system and development goals and in consensus with policy makers" [4]. The establishment of mental health systems research groups within established academic environments that are closely linked to policy development and implementation will strengthen capacity to secure research funds from national and international sources. Support from the IOMHS Technical Reference Group, which will advise on technical aspects of research design, will improve the capacity of such groups to compete for scarce research funds, as will a growing record of publications in international peer-reviewed scientific journals.

## Research governance

Governance of health research determines the means by which stakeholders set, pursue and achieve agreed research goals – what kinds of research should be supported, by whom it should be done and for what ends [16,17]. Among the criteria for governance are representativeness, transparency and accountability. Good research governance is essential to ensure that research is conducted ethically, that it is relevant to the population being researched, and that it is of high scientific quality.

In many low and middle-income countries there is not a formal system in place for review of the ethical dimensions of proposed research projects, whether the research is being conducted by local or international organisations. Where this is the situation the collaborating organisations in which the mental health systems research groups are located will promote and support the establishment of ethics review procedures that protect the rights of all concerned, and ensure the ethical conduct of research and of the use of research findings.

There is little interaction between research groups within and across low and middle-income countries [18]. Most interaction is between research groups in low and middle-income countries and research groups or funding organizations in high-income countries [17]. This is a situation that must be changed. Interaction between organisations within and across low and middle-income countries must be strengthened. Good research governance is particularly important in circumstances where collaborative research is being carried out, particularly collaboration between researchers in low and middle-income countries and high-income countries. The collaboration must be equal, with the interests and health needs of the population in the low and middle-income countries being of paramount importance.

## Summary

The International Observatory on Mental Health Systems is:

1. The mental health systems research, education and development groups;

2. The IOMHS Steering Group;

3. The IOMHS Technical Reference Group; and

4. The IOMHS Secretariat.

The International Observatory on Mental Health Systems will strengthen capacity for mental health systems research, and the translation of that research into benefit for people with mental illness, in low and middle-income countries. Success will be dependent on free and open collaboration, sharing of knowledge and skills, and governance arrangements that are inclusive and that put the needs and interests of people with mental illness and their families at the centre of decision-making. It will also crucially depend on a collective capacity to mobilise resources that will support the work of the Observatory.

We welcome contact from individuals and organisations who wish to contribute to achieving the goals of the Observatory. We would particularly welcome contact from academic and other institutions that may wish to establish a mental health systems research, education and development group and to become active collaborators in the IOMHS network.

## Competing interests

The author declares that they have no competing interests.
